# Current approaches to diagnosing and treating idiopathic granulomatous mastitis: A summary from in-depth clinician interviews

**DOI:** 10.1016/j.heliyon.2024.e38345

**Published:** 2024-09-24

**Authors:** Seeu Si Ong, Jean Xiang Ying Sim, Ching-Wan Chan, Peh Joo Ho, Zi Lin Lim, Mikael Hartman, Jingmei Li

**Affiliations:** a Genome Institute of Singapore (GIS), Agency for Science, Technology and Research (A∗STAR), Singapore, 138672, Singapore; b Department of Surgery, Yong Loo Lin School of Medicine, National University of Singapore and National University Health System, Singapore, 119228, Singapore; c Department of Infectious Diseases, Singapore General Hospital, Singapore, 169608, Singapore; d Department of Infection Prevention & Epidemiology, Singapore General Hospital, Singapore, 169608, Singapore; e Department of Surgery, National University Hospital and National University Health System, Singapore, 119228, Singapore; f Solis Breast Care & Surgery, Mount Elizabeth Medical Centre, Singapore, 228510, Singapore; g Saw Swee Hock School of Public Health, National University of Singapore and National University Health System, Singapore, 117549, Singapore

**Keywords:** Idiopathic granulomatous mastitis, diagnosis, Histopathology, Corticosteroids, Methotrexate, Clinician interviews, Healthcare practices, Breast health

## Abstract

**Background:**

Idiopathic granulomatous mastitis (IGM) is a rare, chronic inflammatory breast condition primarily affecting women of reproductive age. Its diagnosis is challenging due to similarities with other breast disorders, necessitating exclusion of other granulomatous diseases. The management of IGM remains inconsistent and unclear, with high recurrence rates and varying practices.

**Methods:**

This qualitative study involved semi-structured interviews with nine clinicians from Singapore, Malaysia, and Egypt to examine current diagnostic and therapeutic approaches for IGM. Transcripts were analysed using NVivo software for coding and summarisation.

**Findings:**

Clinicians predominantly used imaging and histopathology for diagnosis. Treatment commonly involved corticosteroids, though dosages and tapering regimens varied widely. Methotrexate was used sparingly for refractory cases due to associated risks. Surgical interventions were infrequent, reflecting a preference for medical management. There was a consensus on the need for randomised controlled trials (RCTs) to establish standardised treatment protocols.

**Interpretation:**

This study reveals the complex nature of IGM diagnosis and treatment from clinicians in Singapore, Malaysia and Egypt. This underscores the need for more specific and definitive diagnostic tests, rather than relying on exclusionary methods, and standardised treatment guidelines. Multi-centre RCTs are essential for developing evidence-based protocols to improve patient outcomes and address regional differences effectively.

## Highlights

• IGM diagnosis relies on imaging and histopathology.
• Corticosteroids are the primary treatment for IGM.
• Methotrexate is rarely used, and only for refractory cases.
• Surgery is infrequently employed in IGM management.
• Clinicians emphasise the need for RCTs to standardise IGM treatment.

## 1 Introduction

Idiopathic granulomatous mastitis (IGM) is a rare, chronic inflammatory breast disease characterised by the formation of non-caseating granulomas within the mammary tissue [1,2]. The aetiology of IGM is ambiguous, potentially involving autoimmune processes, localised infections, hormonal imbalances, and genetic predispositions [[1], [2], [3], [4]]. Affecting primarily women of reproductive age, IGM often mimics breast carcinoma and infection such as breast abscesses, presenting with palpable masses, pain, erythema, and sometimes nipple retraction or discharge [5]. This clinical overlap poses significant diagnostic and therapeutic challenges.

Diagnosis of IGM is primarily one of exclusion, requiring the thorough elimination of other granulomatous diseases such as tuberculosis, fungal infections, and sarcoidosis [6]. Diagnostic approaches include clinical examination, imaging techniques, microbiological cultures and histopathological evaluation [1,2,4,7]. Although imaging modalities like mammography and ultrasound are commonly used, they often yield nonspecific results [8,9]. Histopathological examination is essential, as it reveals granulomas without caseous necrosis and chronic inflammatory infiltrates [2,5]. Typically, cultures for bacteria, fungi, and mycobacteria are negative, and systemic granulomatous diseases are absent, supporting the IGM diagnosis [4,6]. Some studies have highlighted the association of Corynebacterium species, particularly C. kroppenstedtii, with IGM, suggesting its potential role in the disease's pathology [4,[10], [11], [12], [13], [14]].

Management of IGM is complex and diverse, ranging from conservative treatments with antibiotics and corticosteroids to surgical interventions like abscess drainage, wide local excision, and mastectomy [15]. Corticosteroids, though commonly used, have significant side effects and high recurrence rates [15,16]. Methotrexate, a non-steroidal immunosuppressive agent, has been explored as a treatment option with mixed success [15,[17], [18], [19], [20]]. Published literature also describes corticosteroids and methotrexate being administered with variable initial dosages and tapering regiments [15,16,21]. Surgical options are reserved for refractory or recurrent cases [22]. The high recurrence rates and inconsistent treatment approaches and responses highlight the need for further research to establish more effective management strategies [15].

Geographical variations in IGM incidence and management have been noted, with higher prevalence in the Middle East, Central Asia, and East Asia [10,15]. This could potentially be attributed to genetic, environmental, or clinical practice differences. Understanding these regional practices and challenges is beneficial for developing tailored guidelines to improve patient outcomes.

The study aims to provide an in-depth overview of the clinical management strategies used for diagnosing and treating IGM. It seeks to identify consensus and discrepancies in practice, uncover regional variations, and highlight the potential benefits and challenges of conducting randomised controlled trials (RCTs) for IGM treatments. By synthesising expert insights, this study aims to contribute to a better understanding of IGM management, potentially guiding future research and clinical practice in this challenging field. The findings will contribute to the development of guidelines for effective IGM management and help inform future research directions.

## 2 Methods

### 2.1 Study design

This qualitative study employs a research design of in-depth interviews with clinicians in Singapore, Malaysia, Indonesia, and Egypt. The primary aim is to discuss the current practices and challenges in diagnosing and treating IGM. Ethical approval was obtained from the Institutional Review Board (IRB) of the National University of Singapore (Reference code: NUS-IRB-2020-653). Informed consent was obtained from all participants, ensuring they understood the study's purpose, the voluntary nature of participation, and their right to withdraw at any time without consequence. Participants were assured of the confidentiality and anonymity of their responses. The study adhered to the principles outlined in the Declaration of Helsinki.

### 2.2 Participant selection

Clinicians practising in major hospitals in Singapore, Malaysia, Indonesia, and Egypt were identified through professional contacts and referrals (i.e., snowball sampling). The inclusion criteria were:

● Experience in diagnosing, treating, or managing patients with IGM.
● Willingness to participate in a detailed interview regarding their clinical practices.

### 2.3 Data collection

Data were collected through semi-structured interviews conducted either in person or via video calls, depending on the participant's preference and availability. An interview guide was developed by SSO based on existing literature and expert consultations with CWC, MH and JL, covering the following topics:

● Diagnostic criteria and investigations for IGM.
● Treatment protocols and preferences.
● Challenges encountered in diagnosis and treatment.
● Potential benefits and feasibility of conducting an RCT for IGM treatments.

A sample of the questions included in the interview guide can be found in Supplementary Appendix A.

### 2.4 Interview process

Each interview lasted approximately 60–90 min and was audio-recorded with the participant's consent. Interviews were conducted in English, and any specific medical terms or protocols mentioned were clarified during the interview to ensure accurate understanding and recording. Field notes were also taken to capture contextual information.

### 2.5 Data analysis

The audio recordings were transcribed verbatim, and the transcripts were then subjected to a coding process to summarise the interview data. This process involved identifying key topics and patterns across the responses, allowing for the extraction of significant insights regarding the diagnosis and treatment of IGM. NVivo software (version 12) was used to facilitate the coding process. The main steps involved in the summarisation process were:

● Transcription: Converting audio recordings into text with *Whisper.* [23].
● Initial Reading: SSO read through the transcripts to get an overall sense of the content.
● Coding: SSO, JXYS and MH performed systematic coding of the transcripts to identify significant points related to diagnostic criteria, treatment protocols, challenges, and opinions. The coding process organised the data into manageable segments for analysis.
● Summarisation: SSO summarised the coded data to extract key findings. The summarisation focused on highlighting common practices, challenges faced by the clinicians, and their insights on the management of IGM.
● Organising Information: SSO structured the summarised information into categories reflecting the interview guide topics, ensuring a comprehensive representation of the participants' views.

To ensure the reliability and validity of the findings, several strategies were employed:

● Triangulation: SSO compared the findings with existing literature and discussed emerging insights with the secondary coder, JXYS.
● Member Checking: Providing participants with a summary of the findings to verify the accuracy and interpretation of their responses.
● Peer Debriefing: SSO held sessions with PJH, ZLL and JL to review and critique the analytical process.
● Reflexivity: SSO maintained a reflexive journal to document thoughts, assumptions, and potential influences on the research process.

The research was funded for the open access journal publication fee, with no influence from the funding source on study design, data collection, analysis, or publication decisions.

## 3 Results

### 3.1 Participant characteristics

21 clinicians were approached through professional contacts. One clinician who did not fulfil the inclusion criteria, referred three other clinicians. Two clinicians were further recommended by consented participants (snowballing). Of the 26 clinicians approached to be included in the study,

● No response: 12
● Did not fulfil inclusion criteria: Five
● Included in our study: Nine

In-depth interviews were conducted according to the interview guide (Appendix A) with the 9 clinician participants who provided written, informed consent. Recruited participants have a combined 155 years of surgical clinical experience (median 16 years, range 2–32 years). Participant characteristics are shown in Table 1.

**Table 1:** Participant characteristics (n = 9).

Characteristics (n = 9)	*n* (%)
**Years of experience as breast surgeons, median [range]**	16 [2–32]
**Country of clinical practice**	
Singapore	5 (55.5)
Malaysia	3 (33.3)
Egypt	1 (11.1)
**Site of practice**	
Public hospital	7 (77.7)
Public and private hospital	2 (22.2)

### 3.2 Themes and sub-codes identified from interview transcripts

Four main themes were covered in the interviews: Clinical prevalence and patient presentation; Diagnosis of IGM; IGM treatment approaches; and Conducting an RCT for IGM treatments. The sub-codes, definitions and example quotes for the four themes can be found in Supplementary Appendix B.

### 3.3 Clinical prevalence and patient presentation

Most clinicians who practiced in public hospitals only reported seeing around three to six IGM patients in the year before the interview was conducted. This number was 15 and 20, for the two clinicians who practised in both public and private healthcare facilities. Of these, 90 % and 80 % of the cases, respectively, were seen in their private practice. Eight clinicians (8/9, 88.8 %) recalled that majority of their cases were new patients, the remaining clinician (1/9, 11.1 %) reported majority recurrent patients.

**Fig. 1:**
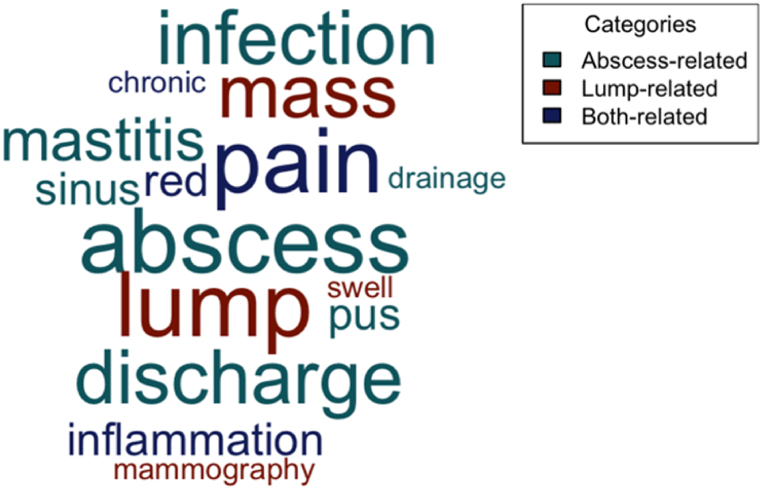
Word cloud of IGM patients' presentations in the clinic. The size of the word corresponds to the frequency in clinicians' response for describing IGM patients' presentation in clinic; the colour corresponds to words used to describe breast abscess, breast lump, or both.

Patients would often present with either a breast lump, or an abscess of the breast, or both. A word cloud of the clinicians’ responses for patient presentation was generated (Fig. 1). Across all 9 clinicians, “abscess” and “lump”, were used with equal frequency (Fig. 1).

### 3.4 Diagnosis of IGM

**Fig. 2:**
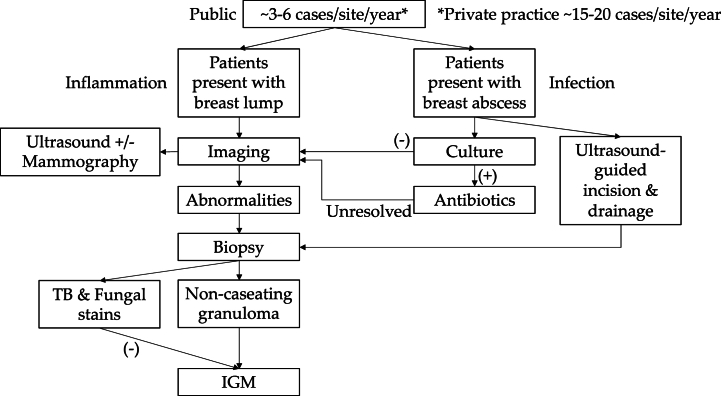
IGM diagnostic workflow described by clinicians.

There was consensus across responses from all clinicians for achieving a clinical diagnosis of IGM (Fig. 2). Briefly, the clinical workflow is as described below. Patients who present with breast abscesses would have pus sent for bacterial culture. Non-purulent patients will not have any samples cultured. Sample retrieval for culture is performed through the following modalities:

● Pus swab
● Percutaneous drainage of abscess
● Ultrasound-guided incision and drainage, usually only for large abscesses.

Patients with culture-positive samples are treated with antibiotics. Patients who are not responsive to antibiotics or have culture-negative samples are further investigated with imaging and biopsy. All clinicians (9/9, 100.0 %) use ultrasound imaging. Three clinicians (22.2 %) perform both ultrasound and mammography in all suspected cases; 1 clinician (11.1 %) will also perform dual imaging but only in women above age 40 for breast cancer concerns. Abnormalities observed from imaging are biopsied. Large breast abscesses undergoing ultrasound-guided incision and drainage are biopsied at the same time. IGM diagnosis is achieved when non-caseating granulomas are observed in histopathology, along with negative stains for tuberculosis and fungus, as well as clinical examination and detailed medical history that contributes to the exclusion of relevant IGM mimics. The diagnostic work-up is summarised in Fig. 2.

### 3.5 IGM treatment approaches

#### 3.5.1 Antibiotics and abscess drainage

As described above, the investigative work-up to a definitive IGM diagnosis is long. Before IGM diagnosis is confirmed, patients are treated as the more common breast abscess. Pre-diagnosis treatment often involves antibiotics, and drainage for large abscesses:

“Most of the time when they come in, they end up having surgery because they come in fresh with a large abscess. So usually, you go in to drain the abscess, you do biopsy, but then you realise it's IGM.” – Clinician 1

“I think that would be about a quarter of our patients would have been partially treated elsewhere, either with antibiotics or with repeated incisions” – Clinician 2

“If we think it's a breast abscess, then commonly what is done is that we drain it percutaneously, send it for culture to see if there's any bacteria and we can give them a short course of antibiotics.” – Clinician 3

Prior to diagnosis of IGM, beta-lactams (penicillin) was the main antibiotic class utilised for empirical therapy (9/9, 100.0 %). Of these, 7 (77.7 %) use amoxicillin, of which 2 (22.2 %) use amoxicillin-clavulanate; 1 (11.1 %) uses cloxacillin; 1 (11.1 %) unspecified. Post-diagnosis treatment allocated is predominantly steroids:

“[When] biopsy comes back as confirmatory of IGM, then yeah, I'll start prednisolone.” – Clinician 4

“Once the diagnosis comes out for IGM, we will switch to steroids.” – Clinician 2

“The biopsy confirmed is IGM, so, so easy, we can start steroid[s] and then taper it down.” – Clinician 5

“Let's say biopsy comes back, it's clearly stated as the granulomatous mastitis, um, then that's the time that we counsel the patient to start with steroids.” – Clinician 6

#### 3.5.2 Prednisolone

Eight clinicians (88.8 %) choose steroid treatment (prednisolone) for first-line treatment (Supplementary Appendix B) after IGM diagnosis is confirmed, with varying starting, maintenance, and tapering regimens (Table 2).

**Table 2:** Steroid dosages reported by the 7 clinicians with prednisolone as first-line treatment after IGM diagnosis confirmation.

**Clinician participant**	**Starting dose (daily), mg**	**Maintenance**	**Tapering**
Clinician 1	30	Review every 4–6 weeks	Not specified
Clinician 2	10	Review every 6 weeks, for 2–3 months	None
Clinician 3	50	Review every 2 weeks	Taper over 2 months.
Clinician 4	40	Review every 2 weeks	After 2 weeks, decrease to 30 mg/day for 2 weeks, 20 mg/day for 2 weeks, 10 mg/day for 2 weeks.
Clinician 5	60	Review every 2 weeks	With improvement in 2 weeks, continue at 60 mg/day for 2 more weeks, then decrease to 30 mg/day for 2 days, 20 mg/day for 2 days, 10 mg/day for 2 days
Clinician 6	30	Review every 2 weeks	With improvement, decrease by 5 mg/day after 2 weeks, then every month.
Clinician 7	20–30	Review every 2 weeks	Without complete resolution in 2 weeks, continue at initial dose for 2 more weeks. Half the dosage every 3 days.
Clinician 8	Not specified	Review every 2 weeks	Tail down every 2 weeks.

#### 3.5.3 Non-steroidal immunosuppressive methotrexate

The remaining one clinician (11.1 %) treated patients with confirmed IGM diagnosis with immunosuppressive methotrexate.

“We give methotrexate [at 40mg] once per week for three months.” – Clinician 9

Clinician 9 also uses methotrexate in the same regimen, as second-line treatment for patients who are referred as non-responders to prednisolone. The other 8 clinicians have not treated IGM patients with methotrexate; 3 (33.3 %) have referred patients who cannot resolve with steroid treatment, to rheumatology for methotrexate treatment.

“If they do not resolve with prednisolone or if they recur very shortly after stopping the prednisolone, I will, I will refer them to rheumatology.” – Clinician 4

“Methotrexate is definitely not a first-line treatment option, it's more like a if all else fails kind of thing.” – Clinician 3

#### 3.5.4 Spontaneous resolution

Clinicians were asked about their experience with spontaneous resolution of IGM without medical treatment. Five clinicians (55.5 %) have not encountered such patients, 3 clinicians (33.3 %) have had patients with mild disease resolve without medical intervention, and 1 clinician (11.1 %) with a patient who refused steroid treatment with spontaneous disease resolution.

“Do you have any patients who you have treated for IGM with observation only?” “No, patient will be very upset with you.” – Clinician 8

“If they are small and they are not causing problems, I avoid them. The condition seems to clear on its own after a while if it's considerably small.” – Clinician 1

“They refuse[d] prednisolone. Then we just observe them over a period of time. Then for some reason, spontaneously, the inflammation resolves over a matter of a few weeks.” – Clinician 4

#### 3.5.5 Surgical interventions

Seven clinicians (77.7 %) concurred that surgical intervention is not helpful for IGM management.

“Because if there are large areas, it doesn't work. Unless you excise half the breast, the area of damage and inflammation doesn't disappear.” – Clinician 1

“I usually tell patients the surgery is the last resort because it doesn't get rid of problem. I've never done, uh, any surgical procedure for IGM.” – Clinician 4

“There is no role of surgery in management of [IGM]. No surgical treatment at all, no surgical intervention.” – Clinician 9

The remaining 2 clinicians (22.2 %) have performed excisional surgical treatment for IGM patients in rare instances of a recurrent case, and in patients with comorbidities where medical treatment is not suitable.

“It's usually when medical interventions are not working. Or recurrent. It is quite rare to use surgical. I only did [it] once. Excise the area.” – Clinician 5

“For diabetic patients or the immunocompromised patients, then we talk about excision. If the patient is, uh, if it is disturbing the patient too much, and they are keen for excision. I will just, I will offer excision.” – Clinician 7

#### 3.5.6 Follow-up

There are varying practices with regards to follow-up. Two clinicians (22.2 %) follow patients until complete disease resolution, then return patients to national breast cancer screening guidelines mammography timeline.

“So, if there are no more signs of inflammation and there's no evidence of any more collection, patient asymptomatic, then you try and say bye-bye” – Clinician 8

Five clinicians (55.5 %) continue patient follow-up for another 6 months to 1 year.

“So, most of the time it's resolved from treatment, and they don't relapse after six months to one year after discharge. So, I monitor that for about a year after, no relapse and discharge.” – Clinician 1

“So, based on the last ultrasound they had, its complete resolution, then it’s back to normal screening if you're at a high-risk group, or else maybe just a follow up in six months, without ultrasound or even a year. So, if it's normal and low risk, we [will] probably discharge them after that.” – Clinician 6

### 3.6 Conducting an RCT for IGM treatment

Clinician participants were in consensus on the necessity for an RCT to develop standardised treatment for IGM patients because there are no established clinical guidelines.

“If you just look at IGM treatment from a very general perspective, in a sense that there's no established clinical guidelines when it comes to prescribing.” – Clinician 2

“Well, I think a lot of things, um, still in progress, you know, there's no big studies on what's the best for IGM, and the ideal guidelines are also lacking. What we want is a final guideline, not to say a really, uh, an international kind of guideline, but at least our local population-based guideline.” – Clinician 6

“It's good if you could find an RCT because there's actually no real protocol.” – Clinician 7

Clinicians raised concerns over recruiting enough patients for sufficient power to observe statistical and clinical significance between treatment arms.

“Straight away your problem is going to be sample size.” – Clinician 2

“Not easy because the numbers are very small, and we need to get people to be willing to collaborate because in Singapore I think each centre the most 10 new cases or even lesser a year.” – Clinician 3

“Depending on what are the sample size that you wanted, because if you're looking at, um, a hundred per institution per year, I doubt that it's possible” – Clinician 6

Ethical concerns were also raised over studying methotrexate as a treatment arm.

“I'm not familiar with the complications and long-term monitoring required for methotrexate.” – Clinician 1

“I have no experience in using methotrexate and it's also, you know, immunosuppressant. So, uh, a little bit sceptical in studying that because of concern, um, the ethics concern and whether there'll be any side effects to others.” – Clinician 6

## 4 Discussion

In-depth interviews have revealed both areas of consensus and other areas that show variability in clinical management. There is complexity in managing this challenging condition. The diagnostic approach for IGM among the clinicians interviewed involving initial culture tests for bacterial infections, followed by imaging and biopsy for histopathological confirmation, is corroborated in the published literature [1,2,7]. The consensus on the necessity of histopathological confirmation with the presence of non-caseating granulomas and the absence of other granulomatous diseases aligns with current literature [1,2]. This standard diagnostic workflow ensures that IGM is accurately diagnosed, although the prolonged process may delay definitive treatment initiation. The diagnosis of IGM must move away from one of exclusion, and specific tests must be designed for definitive detection and monitoring.

It is important to note that our study reflects the real-world diagnostic practices of breast surgeons. These surgeons rely on histopathology reports, along with clinical examination, medical history, and results from radiological and microbiological assessments, to make their final diagnosis. Our results are limited by an absence of detailed descriptions of pathological examination, radiological observations, and specific laboratory findings since we interviewed surgeons specifically for their central role in diagnosing and managing IGM. We acknowledge that input from other specialists (e.g., radiologists, pathologists, microbiologists) would have added value to the study. The interviewees also did not go into detailed description of their exclusion of infectious agents, vasculitis, neoplastic processes, and other mimics, before achieving IGM diagnosis. These limitations highlight that a more comprehensive clinico-laboratory approach is needed beyond what is captured in the surgeons' practices. Supplementary Fig. S1 reflects a broader diagnostic process involving the multiple diagnostic pillars for IGM.

The treatment landscape for IGM revealed by this study is less diverse than previously described [15,21,[24], [25], [26]]. The predominant use of antibiotics and abscess drainage as pre-diagnosis treatments underscores the initial clinical suspicion of bacterial abscesses in IGM presentations [1,4,5]. However, once IGM is confirmed, the shift to steroid treatment as the primary modality is evident. The variability in steroid dosages and tapering regimens reflects the lack of standardised guidelines and the clinicians' reliance on empirical evidence and personal experience.

The limited use of methotrexate, reserved primarily for refractory cases or as second-line treatment, reflects the cautious approach of clinicians toward non-steroidal immunosuppressive therapies. This is in line with existing literature that suggests methotrexate can be effective but is associated with significant risks [15,[17], [18], [19], [20]]. Surgical intervention is not the mainstay of therapy. Participants believe that excisional surgical interventions do not address the root cause of IGM. The reluctance to use excisional surgical interventions further emphasises the preference for medical management to avoid disfigurement and complications associated with surgery. Avoidance of surgery is also driven by the desire to preserve breast tissue and appearance, and patient quality of life [1,2,6,22].

Clinician 6's platitude that even a local or region-specific guideline will be helpful, underscores the treatment ambiguity and the reliance on historical clinical experience for treatment decisions. The unanimous agreement among the clinicians on the necessity of conducting an RCT to establish standardised treatment protocols for IGM is a significant finding. The challenges identified, including patient recruitment and ethical concerns regarding treatment arms, are critical considerations for designing such studies. Given the rarity of IGM and the small number of cases seen annually by individual clinicians, multi-centre collaborations will be essential to achieve the necessary sample size for an adequately powered RCT. Full randomisation of treatment allocation is unlikely, given the severity spectrum of the condition. The diversity and heterogeneity of the disease, and the inherent diagnosis complexities of IGM, also add to the challenges of performing an RCT for IGM treatments.

The qualitative nature of the interviews limits the generalisability of the findings. Additionally, the reliance on self-reported practices may introduce recall bias. A key limitation of this study is that seven out of the nine clinicians interviewed have a low annual number of IGM patient assessments, which may limit the generalisability of the findings, given the variability in clinical presentations and treatment responses. However, their clinical experience, despite a low volume of cases, might still provide valuable qualitative insights, especially in rare diseases like IGM. Future studies should aim to include a larger and more diverse sample, particularly clinicians with higher volumes of IGM cases, to enhance the generalisability of findings. In addition, including perspectives from other healthcare professionals, such as radiologists and pathologists, could further enrich the data and provide a more comprehensive understanding of IGM management.

## 5 Conclusions

Amongst the complexity in diagnosing and treating IGM, this study has revealed a strong consensus on diagnostic methods and a predominant reliance on steroid treatments post-diagnosis. The variability in treatment approaches, particularly regarding steroid dosages and the cautious use of methotrexate, underscores the need for informed and effective treatment guidelines. Multi-centre RCTs will be essential to developing evidence-based management protocols for IGM. This work contributes to the foundation for future research and guideline development aimed at improving patient outcomes in the region.

## Funding

The research was funded for the open access journal publication fee, with no influence from the funding source on study design, data collection, analysis, or publication decisions.

## CRediT authorship contribution statement

**Seeu Si Ong:** Writing – original draft, Visualization, Project administration, Methodology, Investigation, Formal analysis, Data curation, Conceptualization. **Jean Xiang Ying Sim:** Writing – review & editing, Methodology, Formal analysis. **Ching-Wan Chan:** Writing – review & editing, Methodology, Conceptualization. **Peh Joo Ho:** Writing – review & editing, Supervision, Methodology. **Zi Lin Lim:** Writing – review & editing, Methodology. **Mikael Hartman:** Supervision, Methodology, Investigation, Funding acquisition, Conceptualization. **Jingmei Li:** Writing – review & editing, Supervision, Project administration, Methodology, Investigation, Funding acquisition, Conceptualization.

## Declaration of competing interest

The authors declare that they have no known competing financial interests or personal relationships that could have appeared to influence the work reported in this paper.
