# Supplementary material for: Current approaches to diagnosing and treating idiopathic granulomatous mastitis: A summary from in-depth clinician interviews
Source: Heliyon. 2024 Sep 24;10(19):e38345. doi: 10.1016/j.heliyon.2024.e38345 (PMC11461993; doi:10.1016/j.heliyon.2024.e38345)
Supplement: Multimedia component 1 [file mmc1.docx]

# SUPPLEMENTARY MATERIALS

**Supplementary Figure S1.** IGM diagnosis based on four pillars of clinical presentation, radiologic findings, microbiological studies, and histopathology.

## Supplementary Appendix A

Idiopathic Granulomatous Mastitis: The Clinician’s Perspective – Interview Questions Guide

Clinical Prevalence

1. How many idiopathic granulomatous mastitis (IGM) patients do you see a year in total?
   1. Prompt: Include newly diagnosed patients, patients continuing treatment, recurring patients etc. Feel free to use a range.

Diagnosing IGM patients

1. What is the framework for your investigative work up for diagnosing IGM?
   1. Prompt: Patients’ presentation, deciding when to perform biopsy for patient.
   2. Sub-question: What conditions and presentations lead to a biopsy and histopathological investigation?
2. Do you perform cultures on patient samples?
   1. What patient samples do you collect?
   2. What cultures do you perform?
   3. What strains do you commonly observe in cultures?
   4. Are antibiotics treatment allocated according to culture outcomes?
3. Are there any patients you suspect with IGM that you do not biopsy?
   1. Sub-question, if “Yes”: How many patients do you suspect with IGM that are not biopsied (in a year)?
   2. Prompt: This can include patients whose breast infections are resolved with antibiotics treatment that are not further investigated or treated for IGM.
4. Do you have any other information on diagnosing IGM patients not previously discussed?

Treating IGM patients

1. Do you prescribe steroid treatment for treating IGM?
   1. Is this as a first-line or subsequent treatment option?
   2. In which patients do you prescribe steroids? In which patients do you not prescribe steroids? Why?
   3. What is your steroid treatment allocation?
      1. Which steroid do you prescribe?
      2. What is your initial dosage and tapering dosage?
      3. How long will the patients be taking steroids at the respective dosages?
2. Do you prescribe antibiotics treatment for treating IGM?
   1. Is this as a first-line or subsequent treatment option?
   2. In which patients do you prescribe antibiotics? In which patients do you not prescribe antibiotics? Why?
   3. What is your antibiotic treatment allocation?
      1. Which antibiotic do you usually prescribe?
      2. What dosage and duration do you prescribe the antibiotics for?
3. Do you offer surgical treatment for treating IGM?
   1. Is this as a first-line or subsequent treatment option?
   2. In which patients do you offer surgery? In which patients do you not offer surgery? Why?
4. What surgical procedures are allocated for IGM treatment?
   1. Incision and drainage, or excision, or other surgical procedures?
   2. How frequently are the surgical procedures offered as a treatment option? (On average in a year)
   3. What are the surgical and cosmetic outcomes? Does this differ by procedure?
5. Do you treat IGM patients with methotrexate?
   1. Is this as a first-line or subsequent treatment option?
   2. In which patients do you prescribe methotrexate as monotherapy? In which patients do you prescribe methotrexate in combination with other treatments?
   3. In which patients do you not prescribe methotrexate? Why?
6. What is your methotrexate prescription protocol?
   1. Is this prescribed directly by yourself, or referred to a rheumatologist?
   2. What is the dosage and duration of treatment?
      1. Does this differ with first-line and subsequent treatments?
      2. Does this differ with monotherapy and in combination with other treatment?
7. Describe any other treatment for IGM patients.
   1. Prompt: E.g. Observation, minimally invasive comprehensive treatment (MICT), Traditional Chinese Medicine (TCM), etc.
8. Do you have any other information on how you allocate treatment for IGM patients not previously discussed?

The Clinician’s Perspective

1. Does the area of treating IGM benefit from a randomised controlled trial (RCT)?
2. We are currently planning to conduct an RCT to explore three different arms of monotherapy as first-line treatment for IGM. The arms will be antibiotics, steroids and methotrexate. The control arm will be antibiotics and steroids combination therapy. Do you have any alternatives and suggestions to designing the arms of IGM treatment for randomisation?
3. What are some concerns you might have with carrying out this RCT in your institution?
4. Do you have any last thoughts to share about diagnosing and treating IGM?

## Supplementary Appendix B

Transcripts codebook

Main themes: **Clinical prevalence & patient presentations**, **Diagnosis of idiopathic granulomatous mastitis (IGM)**, **IGM treatment approaches**, and **Conducting a randomised controlled trial (RCT) for IGM treatment**

1. Clinical prevalence & patient presentations

| **Sub-codes** | **Definition** | **Example quote** |
| --- | --- | --- |
| Frequency of IGM cases | Refers to how often IGM cases are encountered by clinicians. | "We see around five to ten new cases of IGM every year in our clinic." |
| Variations in patient presentation | Describes the differences in how IGM manifests in patients. | "Some patients come in with just a small lump, while others have significant pain and swelling." |
| Estimating IGM disease burden using ICD codes and pathology reports | Discusses the methods used to estimate the prevalence of IGM. | "By reviewing ICD codes and pathology reports, we get a rough estimate of the disease burden." |
| Potentially missed cases | Concerns about cases of IGM that may go undiagnosed. | "There are likely many cases that we miss, especially those presenting with less severe symptoms." |

1. Diagnosis of IGM

| **Sub-codes** | **Definition** | **Example quote** |
| --- | --- | --- |
| Diagnosis procedures (not including biopsies) | Methods used to diagnose IGM before biopsy confirmation. | "We often start with an ultrasound and mammogram to rule out other conditions." |
| *Sub-code: Observations from cultures* | *Findings from microbiological cultures related to IGM diagnosis.* | *"Cultures rarely grow any bacteria, which helps us lean towards a diagnosis of IGM."* |
| Biopsy and histopathological investigation | The role of biopsy and histopathology in confirming IGM. | "A core needle biopsy revealing non-caseating granulomas is key for diagnosis." |

1. IGM treatment approaches

| **Sub-theme** | **Definition** | **Example quote** |
| --- | --- | --- |
| Pre-diagnosis treatment | Treatments administered before a definitive IGM diagnosis. | “We often start with broad-spectrum antibiotics while awaiting biopsy results." |
| Post-diagnosis treatment | Treatments given after confirming IGM. |  |
| *Sub-code: First-line* | *Initial treatment administered.* | *“Steroids are typically our first-line treatment once IGM is confirmed.”* |
| *Sub-code: Second-line* | *Alternative treatments when first-line treatments fail.* | *“If steroids aren’t effective, we consider methotrexate as second-line option.”* |
| Antibiotic treatment | Use of antibiotics in managing IGM, including specifics on dosage, types of antibiotics used, duration, side effects, and patient profiles where antibiotics are indicated or contraindicated. | "We sometimes use antibiotics to manage secondary infections, typically starting with broad-spectrum antibiotics like amoxicillin-clavulanate at a dosage of 875 mg/125 mg twice daily for 7 to 14 days. However, prolonged use can lead to gastrointestinal side effects such as diarrhoea, and we avoid antibiotics in patients with known allergies or those with recurrent infections where resistance might be a concern." |
| Steroid treatment | Administration of corticosteroids to treat IGM, including details on dosage, tapering schedules, side effects, and patient co-morbidities or lifestyle where steroid treatment is suitable or unsuitable. | "Steroid therapy can reduce inflammation significantly, with a typical starting dose of 40 mg of prednisone daily. The dose is then gradually tapered over several weeks to months. However, long-term use can lead to side effects such as weight gain, hypertension, and osteoporosis. We are cautious in prescribing steroids to patients with diabetes or those at high risk for osteoporosis." |
| Surgical treatment | Surgical interventions employed in treating IGM, covering the types of surgical procedures, indications, disease severity, cosmetic outcomes, postoperative care, and potential complications. | "For abscesses or recurrent cases, surgical excision might be necessary. Procedures range from simple incision and drainage to wide local excision of the affected tissue. Surgery is typically reserved for patients who do not respond to medical management or have recurrent abscesses. Postoperative care includes wound care and monitoring for infection, with complications such as scarring and changes in breast contour being potential concerns. We consider the severity of the disease and aim to balance effective treatment with preserving cosmetic outcomes as much as possible." |
| Methotrexate treatment | Use of methotrexate in IGM treatment, including specifics on dosage, administration routes, monitoring for side effects, patient characteristics where methotrexate is preferred or avoided, and disease severity. | "Methotrexate is effective for patients who don't respond well to steroids, usually administered at a dose of 15 to 25 mg weekly, either orally or subcutaneously. Patients are monitored for side effects such as liver toxicity and bone marrow suppression, which necessitates regular blood tests. Methotrexate is avoided in patients with liver disease or those planning to conceive, given its teratogenic potential. It is particularly useful in severe cases where steroids alone are insufficient." |
| Observation | Monitoring the patient without immediate active treatment. | "In some cases, we opt for observation to see if the condition resolves on its own." |
| Non-conventional treatments | Alternative treatments outside the standard medical practices. | "Some patients have reported improvement with traditional Chinese medicine, though we don't recommend it." |
| Follow-up | Duration and methods of monitoring patients after treatment. | "Patients are usually followed-up up to a year after disease resolution." |

1. Conducting an RCT for IGM treatment

| **Sub-theme** | **Definition** | **Example quote** |
| --- | --- | --- |
| Necessity for conducting an RCT | The need for randomised controlled trials for treatments to manage IGM. | "An RCT could provide the evidence we need to establish standardised treatment guidelines." |
| RCT design | Considerations in designing RCT for IGM | “The RCT should be multi-centre to ensure diverse patient representation.” |
| Concerns with conducting an RCT | Potential issues related to conducting an RCT |  |
| *Sub-code: Ethical concerns* | *Ethical issues* | *"There are ethical concerns about using methotrexate as an intervention arm."* |
| *Sub-code: Feasibility* | *Practical challenges* | *“Recruiting enough participants and ensuring adherence to the protocol are significant challenges.”* |
